# Parental Alcohol Exposures Associate with Lasting Mitochondrial Dysfunction and Accelerated Aging in a Mouse Model

**DOI:** 10.14336/AD.2024.0722

**Published:** 2025-07-20

**Authors:** Alison Basel, Sanat S. Bhadsavle, Katherine Z. Scaturro, Grace K. Parkey, Matthew N. Gaytan, Jai J. Patel, Kara N. Thomas, Michael C. Golding

**Affiliations:** Department of Veterinary Physiology & Pharmacology, School of Veterinary Medicine and Biomedical Sciences, Texas A&M University, College Station, Texas, USA, 77843

**Keywords:** Epigenetics, Mitochondrial Dysfunction, Aging, Stress-Induced Senescence, Paternal Epigenetic Inheritance, Paternal Effect

## Abstract

Although detrimental changes in mitochondrial morphology and function are widely described symptoms of fetal alcohol exposure, no studies have followed these mitochondrial deficits into adult life or determined if they predispose individuals with fetal alcohol spectrum disorders (FASDs) to accelerate biological aging. Here, we used a multiplex preclinical mouse model to compare markers of cellular senescence and age-related outcomes induced by maternal, paternal, and dual-parental alcohol exposures. We find that even in middle life (postnatal day 300), the adult offspring of alcohol-exposed parents exhibited significant increases in markers of stress-induced premature cellular senescence in the brain and liver, including an upregulation of cell cycle inhibitory proteins and increased senescence-associated β-galactosidase activity. Strikingly, in the male offspring, we observe an interaction between maternal and paternal alcohol use, with histological indicators of accelerated age-related liver disease in the dual-parental offspring exceeding those induced by either maternal or paternal alcohol use alone. Our studies indicate that chronic parental alcohol use causes enduring mitochondrial dysfunction in offspring, resulting in a reduced NAD+/NAHD ratio and altered expression of the NAD+-dependent deacetylases SIRT1 and SIRT3. These observations suggest that some aspects of FASDs may be linked to accelerated aging due to programmed changes in the regulation of mitochondrial function and cellular bioenergetics.

## INTRODUCTION

Aging is a natural phenomenon characterized by a progressive decline in cellular function, leading to an increased risk of disease and death [1, 2]. External stressors, including environmental pollution, chronic stress, and long-term drug use, can accelerate biological aging, promoting the onset of age-related adult disease [3]. Although these environmental stressors induce cellular damage and compromise homeostatic repair systems, recent studies indicate that epigenetic mechanisms may also drive the aging process [4, 5]. The emerging roles of epigenetic mechanisms in aging raise the possibility that some aspects of accelerated aging may involve programmed outcomes arising from early life or, potentially, preconception parental exposures.

It is well established that alcohol use disorder is associated with accelerated aging, particularly within the regions of the brain controlling neuromuscular and cognitive function [6-8]. Moreover, researchers consistently demonstrate that adult alcohol use significantly increases DNA methylation-based measures of epigenetic aging [9-18]. However, the capacity of alcohol-induced epigenetic changes to heritably influence cellular aging across the life course or to transmit to offspring remains unknown.

Fetal alcohol spectrum disorders (FASDs) refer to a series of developmental defects and health conditions caused by maternal alcohol use during pregnancy [19]. Clinical studies demonstrate that the life expectancy of individuals diagnosed with FASDs is approximately 42% shorter than the general population [20]. Notably, these studies also reveal that individuals with FASDs display an increased incidence of age-related diseases, including cardiovascular disease, dementia, metabolic abnormalities, hepatic fibrosis, and cancer [21-27]. Consistent with these observations, researchers have found indicators of accelerated epigenetic aging in children with FASDs [28], although not all tests or examined tissues are consistent. Nonetheless, the role of epigenetic aging in the pathogenesis of FASDs and the higher mortality rates observed in this patient population remains unknown.

Although men drink more than women and are more prone to risky consumption patterns [29], FASDs are exclusively attributed to maternal alcohol exposures, and all alcohol messaging exclusively targets women [30]. However, using mouse models, our group and others have found that paternal alcohol use induces FASD-related behavioral, growth, and patterning defects in the next generation [31-34]. In our previous studies, we identified alcohol-induced alterations in sperm-inherited noncoding RNAs and increases in sperm histone 3 lysine 4 trimethylation [35-37], which correlate with programmed alterations in the transcriptional pathways controlling mitochondrial function within the developing offspring [38-40]. In worms, these epigenetic signals heritably influence aging processes and lifespan [4, 5, 41], and mitochondrial dysfunction is an established hallmark of accelerated aging [1, 2]. Notably, in collaboration with the Parnell group, we have also found evidence of altered mitochondrial function arising from gestational alcohol exposures [42].

Despite the established correlation between maternal drinking and paternal alcohol use [43], biomedical studies do not routinely consider male drinking and, therefore, do not consider potential interactions between maternal and paternal alcohol use. Consequently, we do not know if male alcohol use exacerbates FASD outcomes caused by maternal exposures. Our group recently developed a multiplex mouse model [44, 45], enabling us to compare outcomes induced by maternal, paternal, and dual-parental alcohol exposures across the life course. Here, we employed this multiplex model to determine the impacts of preconception paternal and periconceptional maternal alcohol exposures on established measures of cellular senescence and mitochondrial function in middle life. Our experiments reveal that chronic parental alcohol exposure is associated with a lasting deficit in mitochondrial function that correlates with increased markers of premature cellular senescence, hepatic fibrosis, and systemic inflammation. Notably, some of these changes display additive increases in the male offspring of dual-parental alcohol exposures.

## MATERIALS AND METHODS

### Study Approval

We conducted all experiments following the procedures outlined under AUP IACUC 2023-0186, which was approved by the Texas A&M University IACUC. We performed all experiments following IACUC guidelines and regulations. Here, we report our data following the ARRIVE guidelines.

### Animal husbandry and alcohol treatments

We implemented the multiplex 2x2 dual-parental alcohol exposure model using the procedures published previously [44] (Supplementary Fig. 1). Briefly, we obtained adult C57BL/6J (Strain #: 000664 RRID: IMSR_JAX:000664) mice from the Texas Institute of Genomic Medicine (TIGM) and maintained them in the TIGM facility on a reverse 12-hour light/dark cycle (lights off at 8:30 am) with ad libitum access to a standard breeder diet (catalog# 2019, Teklad Diets, Madison, WI, USA) and water.

On postnatal day 83, we individually caged male mice and allowed them to acclimate to individual housing for one week. As part of our animal husbandry, we introduced extra enrichments to the animal's home cage to mitigate the stress associated with individual housing. Specifically, we added shelter tubes to the males' home cages and provided igloos for the females (catalog# K3322 and catalog# K3570 from Bio-Serv, Flemington, NJ, USA). These enrichments remained with the mice throughout the experimental course, transferring through their weekly cage changes. Beginning on postnatal day 90, we randomly assigned males to either the Control or alcohol (EtOH) treatment groups and exposed them to either the control treatment (water) or 10% ethanol (w/v; catalog# E7023, Millipore-Sigma, St. Louis, MO, USA). As described previously [40], we employed a modified version of the Drinking in the Dark model, which allows mice limited, voluntary access to the Control and EtOH treatments. Briefly, males were allowed access to the Control or EtOH treatments for a four-hour period that began four hours after the initiation of the dark cycle. We ensured identical treatment of Control males by switching between two identical water bottles.

On postnatal day 83, we individually caged female mice and allowed them to acclimate to individual housing conditions for one week. We then randomly assigned postnatal day 90 females to the experimental (EtOH) (10% ethanol w/v) or Control treatment groups. Four hours after the initiation of the dark cycle, we replaced the water bottle of the female's home cage with the appropriate treatment bottle. We allowed female mice voluntary access to the Control or EtOH treatment for four hours and then returned their original water bottle to their home cage. We simultaneously exchanged the water bottles of Control and EtOH-exposed dams to ensure identical conditions.

At the end of each week, we recorded the weight of each mouse (g) and the amount of fluid they had consumed (g) and then calculated the weekly fluid consumption as grams of fluid consumed per gram of body weight. To remain consistent with clinical studies, we converted this number to grams per kilogram (g/kg), as described previously [46].

We initiated maternal exposures ten days before breeding dams to treated males. After seven to ten days of exposure, we synchronized female reproductive cycles using the Whitten method [47]. Then, after the daily Control or EtOH treatments, we placed a single female into the home cage of a treated male to generate offspring from control, maternal, paternal, and dual-parental exposures. After six hours, we confirmed matings by the presence of a vaginal plug and returned the female mice to their home cage. We ensured males rested for at least 72 hours before a subsequent mating. We subjected dams to minimal handling and maintained the EtOH and Control treatments until gestational day 10.5. We used a body weight gain of approximately 1.8g to diagnose pregnancy. Upon pregnancy diagnosis on gestational day 10.5, we ceased the Control and EtOH treatments and provided dams with three nestlets, one Manzanita wood gnawing stick (catalog# W0016, Bio-Serv, Flemington, NJ, USA), and one gummy bone (catalog# K3585, Bio-Serv, Flemington, NJ, USA). We then left pregnant females undisturbed with no handling or cage changes and allowed them to deliver their offspring. We resumed cage changes on postnatal day seven, maintaining the cage enrichments (one igloo, three extra nestles, one Manzanita wood gnawing stick, and one gummy bone) throughout the weaning period. We monitored pups daily without handling and weaned male and female offspring into separate cages at postnatal day 21. Thereafter, we weighed offspring once a week and provided ad libitum access to food and water (Supplementary Fig. 1).

### Offspring Dissections and Tissue Collection

At 42 weeks of age, we sacrificed the offspring using CO2 asphyxiation followed by cervical dislocation, then obtained Dual-energy x-ray absorptiometry (DEXA) scans using an UltraFocus DXA Faxitron (Tucson, Arizona). We either fixed the collected tissues in 10% neutral buffered formalin (catalog# 16004-128, VWR, Radnor, PA, USA) or snap-froze the samples on dry ice and stored them at -80ºC. We then used the assays and methods detailed by Kohli et al. to assess histological and molecular markers of cellular senescence and biological aging [48].

### Frozen Sectioning and Oil Red Staining

We dissected a central portion of the frozen liver and embedded it in Optimal Cutting Temperature (OCT) compound (Ref 45-83, Sakura Finetek, Torrance, CA, USA). We sectioned the OCT-embedded livers using a microtome-cryostat at 14µm. Then, we stained the liver sections using an Oil-Red-O kit (catalog# ab150678, Abcam, Boston, MA, USA) per the manufacturer's instructions. We randomly selected three areas of the same tissue section and imaged each area using a VS120 Virtual Slide Microscope (Olympus, Waltham, MA, USA).

### Paraffin Sectioning and Sirius Red Staining

We washed the fixed liver samples in PBS and processed them for histological analysis using a TP1020 Automatic Benchtop Tissue Processor. After processing, we embedded the tissue in paraffin and sectioned it using an RM2255 Rotary Microtome. Per the manufacturer's instructions, we stained the liver sections using a Sirius Red kit (ab150681, Abcam, Boston, MA, USA). We randomly selected three areas of the same tissue section and imaged each area using the VS120 Virtual Slide Microscope (Olympus, Waltham, MA, USA).

### Frozen Sectioning and β-gal Staining

We dissected the frozen brains into left and right hemispheres and embedded the right hemisphere in OCT compound (catalog# 4583, Sakura Finetek, Torrance, CA, USA). We sagittally sectioned the OCT-embedded brains using a microtome-cryostat at 10µm. We stained the sections with a β-Galactosidase senescence staining kit (Cell Signaling, 9860, Cell Signaling, Danvers, MA, USA) per the manufacturer's instructions at a pH of 6 and counterstained with Nuclear Fast Red Solution (ab246831, Abcam, Boston, MA, USA). We randomly selected three areas of the same tissue section and imaged each area using the VS120 Virtual Slide Microscope (Olympus, Waltham, MA, USA).

### RNA Isolation and reverse transcriptase quantitative Polymerase Chain Reaction (RT-qPCR)

We isolated RNA from the offspring's liver, kidney, and brain cortex using the RNeasy Plus Mini Kit (catalog# 74136; Qiagen, Germantown, MD, USA) according to the manufacturer's instructions. We assessed RNA purity and concentration using a NanoDrop 2000 Spectrophotometer (Thermo Scientific, Waltham, MA, USA). We seeded approximately 1ug of RNA into a reverse transcription reaction using the High-Capacity cDNA Reverse Transcription Kit (catalog# 4368814; Thermo-Fisher, Waltham, MA, USA). Next, we determined the relative levels of candidate gene transcripts using the AzuraView GreenFast qPCR Blue Mix LR kit (catalog# AZ-2320: Azura Genomics, Raynham, MA, USA). We performed all reactions on a Bio-Rad CFX384 qPCR machine. Primer sequences are in Supplemental Table 2.

### DNA Isolation and qPCR analysis of mitochondrial DNA copy number

We isolated DNA from 15 ug of frozen liver tissue using the HotSHOT method [49] and measured mitochondrial DNA copy number using the AzuraView GreenFast qPCR Blue Mix LR kit. Primer sequences are in Supplementary Table 2.

### Assays and ELISAs

We determined Alanine Transaminase (ALT) and Aspartate Aminotransferase (AST) activity in the liver using an Alanine Transaminase Activity Assay kit (catalog# ab105134, Abcam, Boston, MA, USA) and Aspartate Aminotransferase Activity Assay kit (catalog# ab105135, Abcam, Boston, MA, USA) per the manufacturer's instructions. We measured the liver NAD+/NADH ratio using the NAD/NADH Assay Kit (catalog# ab65348, Abcam, Boston, MA, USA) following the provided protocol. We quantified hepatic SIRT1 and IL-6 levels using the SIRT1 Mouse SimpleStep ELISA and Mouse IL-6 ELISA kits (catalog# ab206983 and ab100712, respectively, Abcam, Boston, MA, USA) per the manufacturer’s instructions. Following the manufacturer-provided protocol, we quantified hepatic malondialdehyde (MDA) levels using the Lipid Peroxidation Assay (catalog# ab118970, Abcam, Boston, MA, USA).

### Protein Extraction and Western Immunoblot Analysis

We extracted proteins from frozen liver samples using RIPA buffer and quantified protein levels using a BCA Protein Assay Kit (catalog# 23227 Thermo Scientific, Waltham, MA, USA). We loaded 20µg of protein on an 8% SDS polyacrylamide gel and transferred proteins onto a PVDF membrane for 2 hours at 60V. We blocked membranes in 5% milk for one hour and then blotted membranes using antibodies recognizing OPA1 (catalog# CST 80471, Cell Signaling, Danvers, MA, USA), OMA1 (catalog# 67449, Protein Tech, Rosemont, IL, USA), SIRT3 (catalog# 10099-1-AP, ProteinTech, Rosemont, IL, USA), and GAPDH (catalog# CST 51745, Cell Signaling, Danvers, MA, USA) at 4^o^C overnight.

We extracted histones using a Histone Extraction Kit (catalog# ab113476, Abcam, Boston, MA, USA) and quantified the isolated protein using BCA Protein Assay Kit - Reducing Agent Compatible (catalog# 2325 Thermo Scientific, Waltham, MA, USA). We loaded 20µg of protein onto a 12% SDS polyacrylamide gel and transferred it to a PVDF membrane for 2 hours at 60V. We blocked membranes in 5% milk for one hour and blotted the membranes using antibodies recognizing H3K9ac (catalog# 07-352, Millipore Sigma, Burlington, MA, USA), H3K27me3 (catalog# ab192985, Abcam, Boston, MA, USA), H3K9me3 (catalog# ab8898, Abcam, Boston, MA, USA), and Total H3 (catalog# Active Motif, 39763, Carlsbad, CA, USA) at 4^o^C overnight.

We washed blotted membranes in TBST for one hour and then added the appropriate secondary antibodies: anti-mouse (Catalog #926-80010, Li-Cor, Lincoln, NE, USA) or Anti-Rabbit (Catalog# 926-80011, Li-Cor, Lincoln, NE, USA). We imaged the blots using the Li-cor chemiluminescence system (catalog # 926-95000, Li-Cor, Lincoln, NE, USA) with the BioRad Chemidoc MP. We performed a densitometric analysis of bands using ImageJ and the normalized band intensity GAPDH or total histone H3.

### Sex as a biological variable

We designed our study to contrast the impacts of maternal and paternal alcohol use on offspring age-related outcomes. Our previous studies reveal that paternal alcohol exposures consistently induce sex-specific changes in offspring fetoplacental growth and patterning, with male offspring exhibiting more severe outcomes than females [39, 40, 44, 50]. Therefore, we separately compared the impacts of parental alcohol exposure on male and female outcomes.

### Data handling and statistical analysis

We subjected all data generated during this study to the data management practices and statistical analyses described previously [44]. Briefly, we recorded our initial observations by hand and then inserted these measurements into Google Sheets or Microsoft Excel. To analyze offspring organ weights, we normalized the values for organ weights to the animal's total body weight at sacrifice. We normalized gene expression values by importing the RT-qPCR replicate cycle threshold (Ct) values for each gene of interest into Microsoft Excel, then normalized these values to the geometric mean of the reference genes *β*-actin and *α*-tubulin, as described previously [48]. For qPCR analysis of mitochondrial copy number, we imported the replicate Ct values for the mitochondrial DLoop region into Excel, then normalized measures to the Ct values of the Tert gene encoded in the nuclear genome, as described previously [51]. We then used the -ΔΔCT method [52] to calculate the relative fold change for each biological replicate. We calculated the total daily caloric intake during gestation by multiplying the grams of food consumed between gestational Days 0 and 10 by the energy density of the supplied diet. We then determined the calories derived from EtOH by multiplying the grams of fluid consumed by 0.1 (10% EtOH) and 7 Kcal/g ethanol. For the analysis of histological stains, including β-Galactosidase, Oil-Red-O, and Sirius Red, we used Photoshop to standardize the levels, parameters, and saturation of each image. For the analysis of β-Galactosidase, we adjusted the blue color of the senescent cells to black, with the surrounding tissue showing gray; for the analysis of Oil-Red-O, we converted the color scheme to black and white, with the red color of lipid droplets adjusted to black and the surrounding tissue showing gray. We then imported the images into ImageJ, used the threshold property to cover the black stain, and excluded the gray area. We then analyzed images for staining intensity and expressed the quantification as a percentage of the total positively stained tissue area to the total area, as described [53]. We averaged three percentages for each sample to obtain the final % of tissue area. Our histological analysis was conducted using sample blinding, but the molecular analysis of tissue samples was not.

For statistical analysis, we transferred the collected datasets into GraphPad Prism 10 (RRID:SCR_002798, GraphPad Software Inc., La Jolla, CA, USA). We analyzed all data sets with statistical significance set at alpha = 0.05. We first employed the ROUT test (Q = 1%) to identify outliers. Next, we verified the normality of the datasets using the Shapiro-Wilk test and verified equal variance using the Brown-Forsythe test. If data passed normality and variance testing (alpha = 0.05), we employed either an unpaired, parametric (two-tailed) t-test or a One-way or Two-way ANOVA. We then used Tukey's post hoc test to compare each treatment or Dunnett’s test to compare all treatments to the Control. If the data failed the test for normality or we observed unequal variance, we ran a Kruskal-Wallis test followed by Dunn's multiple comparisons test or a non-parametric Mann-Whitney test. We present detailed descriptions of each statistical test and sample size for each Figure in Supplementary Table 1.

## RESULTS

### A multiplex mouse model to study the impacts of parental drinking on offspring cellular senescence and age-related phenotypes.

**Figure 1.**
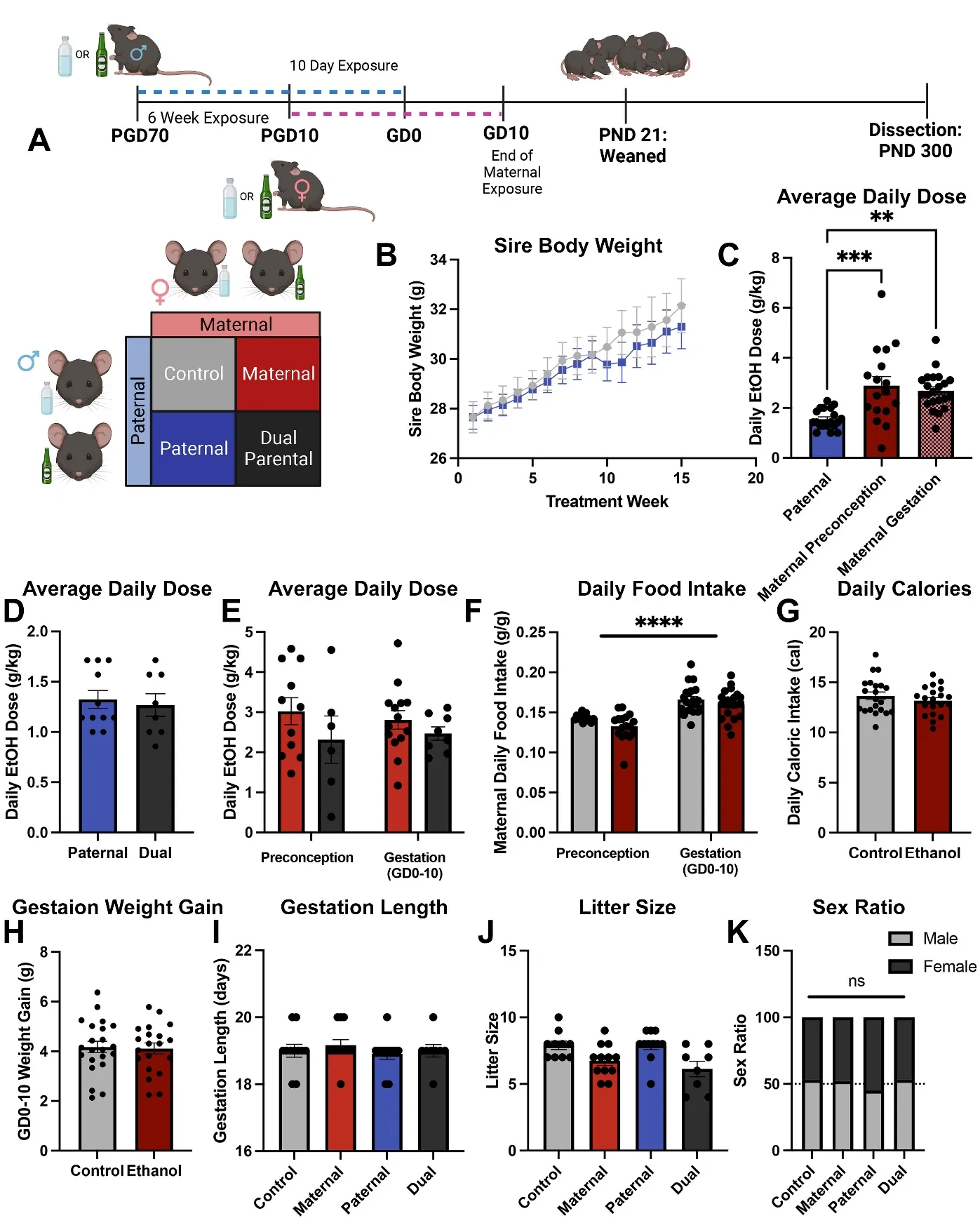
**A multiplex mouse model to study the impacts of maternal, paternal, and dual parental alcohol consumption on offspring premature cellular senescence and accelerated aging**. (**A**) Schematic representation of the experimental design we employed to contrast the impacts of differing patterns of parental alcohol consumption on makers of offspring premature cellular senescence and accelerated biological aging (PGD pregestational day, GD gestational day, PND postnatal day). (**B**) Weight gain of Control (n=16) and EtOH-exposed (n=15) males across the exposure course. (**C**) Average daily dose of EtOH compared between males (n=19) and females and between the preconception (n=17) and gestation phases (n=22). Comparison of average daily EtOH dose between (D) males within the PatExp (n=11) and DualExp (n=8) treatments and (E) females within the MatExp and DualExp treatments during both the preconception and gestational phases. We calculated the average daily dose by multiplying the average weekly fluid consumption (g/g) by 0.10 (10% EtOH), dividing this number by 7 (days), and converting it to g/kg. (**F**) Comparison of daily maternal food intake between the Control and EtOH treatments, both preconception and during gestation (n=13-20). (**G**) Comparison of maternal daily gestational caloric intake, including calories derived from EtOH, between the Control (n=22) and EtOH treatments (n=20). (**H**) Comparison of dam weight gain between gestational days 0 and 10 of pregnancy. Comparison of (I) gestation length, (J) litter size, and (K) the ratio of male and female offspring between the treatment groups (n=8-12). Data represent mean ± SEM, ** P < 0.01, *** P < 0.001, **** P < 0.0001.

We hypothesized that preconception paternal and periconceptional maternal alcohol exposures program a lasting, negative influence on offspring mitochondrial function, leading to markers of stress-induced premature cellular senescence and an accelerated aging phenotype. To test our hypothesis, we employed our recently published mouse model [44], which utilizes a 2x2 factorial experimental design to compare offspring derived from unexposed Controls to maternal (MatExp), paternal (PatExp), and dual parental (DualExp) models of alcohol exposure (Fig. 1A). As both injection and oral gavage induce the systemic stress response, which alters epigenetic programming in sperm and oocytes [54], we utilized a voluntary consumption paradigm where mice consume ethanol (EtOH) according to their individual preference and obtain physiologically relevant plasma alcohol levels, while encountering minimal handling [44].

We did not observe any differences in male weekly weight gain between the Control and EtOH treatment groups (Fig. 1B). EtOH-exposed males received an average daily dose of 1.55g/kg (Fig. 1C), which, in our previous studies, correlated with plasma alcohol concentrations of ~120mg/dL and induced programmed alterations in offspring placental growth and patterning [40]. After six weeks of exposure (approximately one complete spermatogenic cycle), we bred Control and EtOH-exposed males to Control or EtOH-exposed females (Fig. 1A). As most women cease alcohol consumption after becoming pregnant [55], we exposed female mice to EtOH for an initial preconception period, approximately 7-14 days, but ceased exposure after pregnancy diagnosis on gestational day ten.

We did not observe any significant differences in the maternal average daily EtOH dose between the preconception and pregnancy phases (2.68 and 2.8g/kg); however, EtOH-exposed females received a higher average daily dose than EtOH-exposed males (Fig. 1C). We did not identify any differences in maternal average daily EtOH dose between the MatExp or DualExp treatment groups, nor in paternal average daily EtOH dose between the PatExp or DualExp treatment groups (Fig. 1D-E).

We did not observe any treatment effects on maternal daily food intake. Still, as expected, we did observe increased food consumption during pregnancy compared to the preconception window (Fig. 1F). We did not observe any differences in maternal gestational daily caloric intake between treatments (Fig. 1G) nor any impacts of EtOH on maternal weight gain during pregnancy (Fig. 1H). Pair-feeding is an additional treatment often employed to account for altered maternal caloric intake and nutritional deficits when drug exposures reduce maternal food intake during pregnancy [56, 57]. However, consistent with our previous studies [44], maternal EtOH exposure did not impact food consumption or maternal weight gain, and we did not observe any differences in gestational daily caloric intake between treatments. Therefore, we did not implement a pair-fed control.

After diagnosing pregnancy on gestational day ten, we ceased all treatments and animal handling and allowed dams to deliver their offspring. Although obtaining pregnancies in the DualExp group was more challenging, we did not observe any treatment effects on gestation length, litter size, or offspring sex ratio between the experimental treatments (Fig. 1I-K). We randomly selected two to four male and female offspring from each litter for our aging studies (Supplementary Table 1).

**Figure 2.**
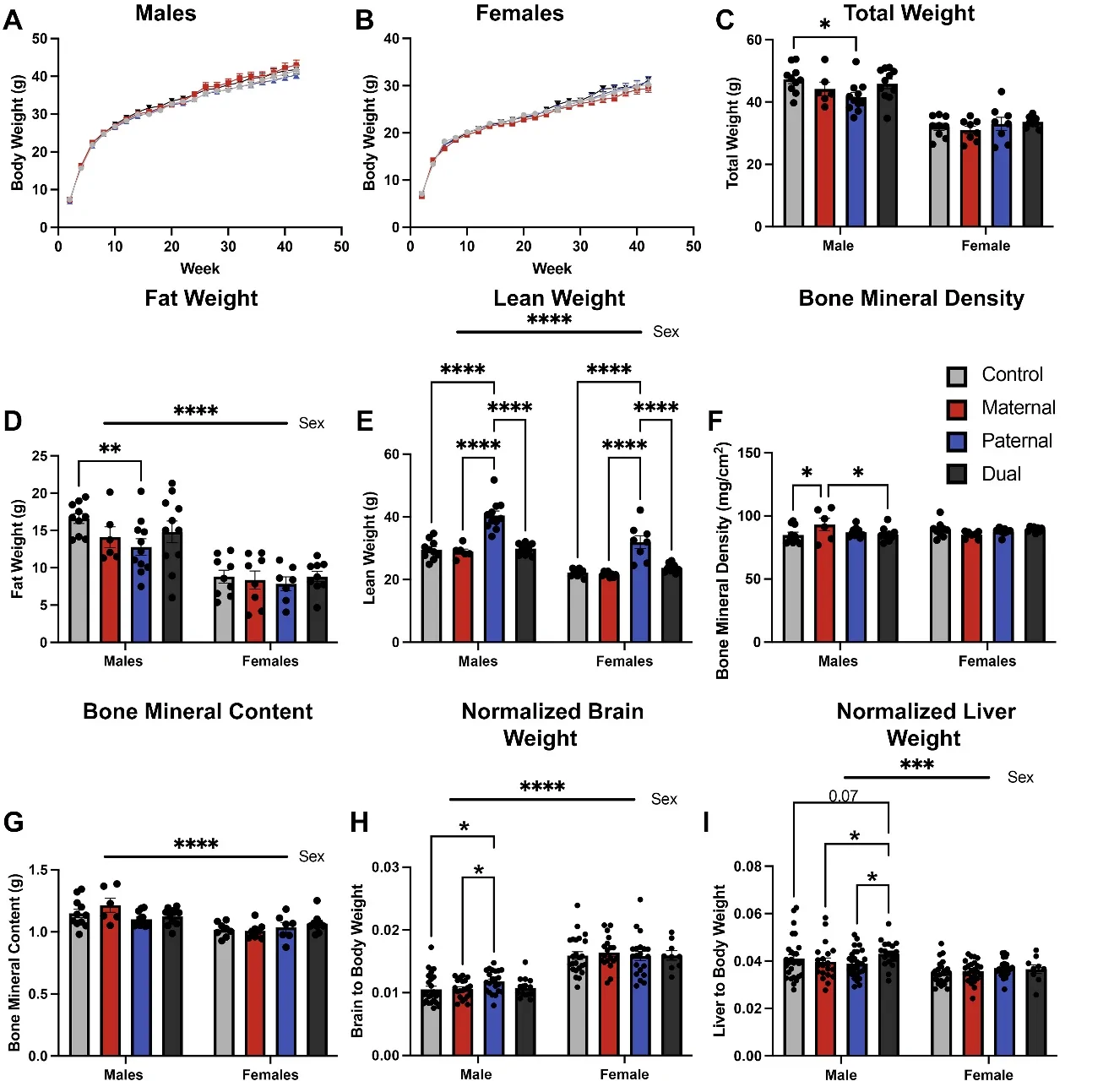
**Maternal, paternal, and dual parental alcohol consumption exert sex- and treatment-specific effects on offspring growth and normalized organ weights**. Comparison of (**A**) male and (**B**) female body weights over 42 weeks of life. On postnatal day 300, we sacrificed the offspring and used Dual-energy x-ray absorptiometry (DEXA) to compare (**C**) total weight, (**D**) fat weight, (**E**) lean weight, (**F**) bone mineral density, and (**G**) bone mineral content between the treatment groups. We compared normalized (**H**) brain and (**I**) liver weights between the treatment groups. We analyzed datasets using repeated measures ANOVA or a two-way ANOVA followed by Tukey's post hoc test. Data represent mean ± SEM, n=9-28, * P < 0.05, ** P < 0.01, *** P < 0.001, **** P < 0.0001.

### Parental alcohol exposures induce sex- and treatment-specific effects on offspring lean weight and normalized organ weights.

In these initial studies, we elected to examine offspring at postnatal day 300 (ten months), representing middle age, a period enabling us to determine if parental alcohol exposures influence the emergence of age-related changes in cellular senescence [58]. We did not observe any differences in offspring growth between treatment groups (Fig. 2A-B); however, at sacrifice on day 300, Dual-energy x-ray absorptiometry (DEXA) scanning identified significant decreases in the total weight and fat weight of PatExp male offspring and consistent with our studies of postnatal day 140 offspring [59], increased lean weights of PatExp male and female offspring (Fig. 2C-E). We identified increased bone mineral density in MatExp male offspring but no treatment effects on bone mineral content (Fig. 2F-G). We did not identify any treatment effects on bodyweight-normalized adrenal, heart, kidney, pancreas, spleen, testis, or thymus weights (Supplementary Fig. 2A-G). However, we did observe increased bodyweight-normalized brain weights in PatExp male offspring and a modest (p=0.07) increase in normalized liver weights in DualExp male offspring (Fig. 2H-I).

### Increased markers of premature cellular senescence in the brains of offspring derived from alcohol-exposed parents.

**Figure 3.**
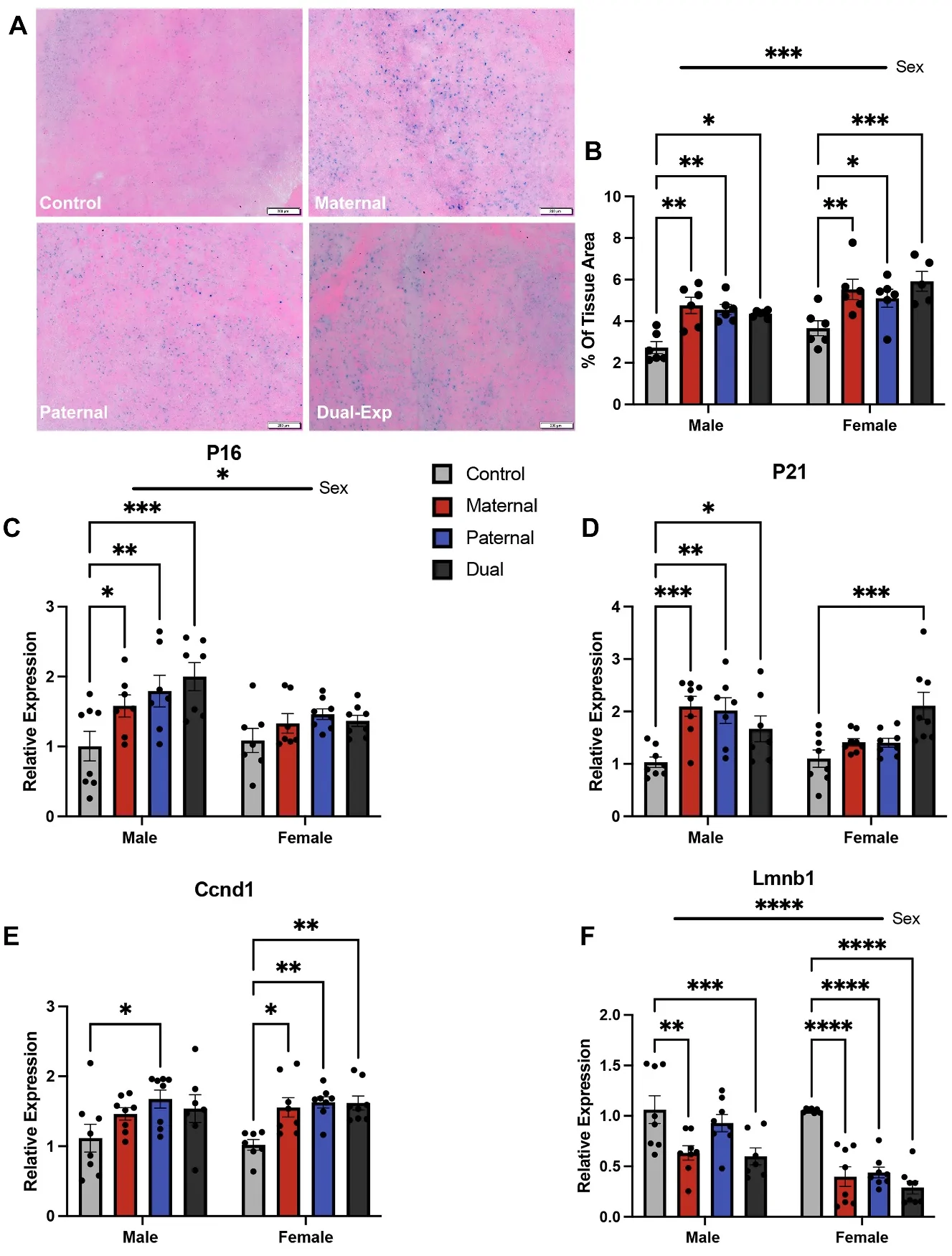
**Maternal, paternal, and dual parental alcohol consumption induce markers of premature cellular senescence in the postnatal day 300 offspring brain**. (**A**) Representative images of female histological sections stained for senescence-associated β-galactosidase (SA-β-gal) activity. (**B**) Quantification of SA-β-gal activity compared between treatment groups. We used reverse transcriptase quantitative polymerase chain reaction (RT-qPCR) to compare transcripts encoding (**C**) p16, (**D**) p21^Ink4a^, (**E**) Cyclin D1 (Ccnd1), and (**F**) *Lamin-B1* (Lmnb1) between treatments. We used a two-way ANOVA followed by Tukey's post hoc test to compare treatment groups. Data represent mean ± SEM, n=6-8, * P < 0.05, ** P < 0.01, *** P < 0.001, **** P < 0.0001.

The brain is particularly vulnerable to oxidative stress and functionally related cognitive decline [60]. Senescent neurons exhibit increased senescence-associated β-galactosidase (SA-β-gal) activity and an upregulation of cell-cycle inhibitory proteins, including p21 and p16^Ink4a^ [48]. We identified increased SA-β-gal staining in the brains of male and female offspring across the MatExp, PatExp, and DualExp treatment groups (Fig. 3A-B). Notably, we identified overall higher SA-β-gal staining in female offspring compared to males. Using RT-qPCR, we identified increases in transcripts encoding the cell cycle genes p21, p16^Ink4a^, and Cyclin D1 (Ccnd1), as well as decreases in the senescence marker *Lamin-B1* (Lmnb1) [48] (Fig. 3C-F).

### Parental alcohol exposures program cumulative effects on the male offspring’s predisposition to develop senescence-associated liver disease.

**Figure 4.**
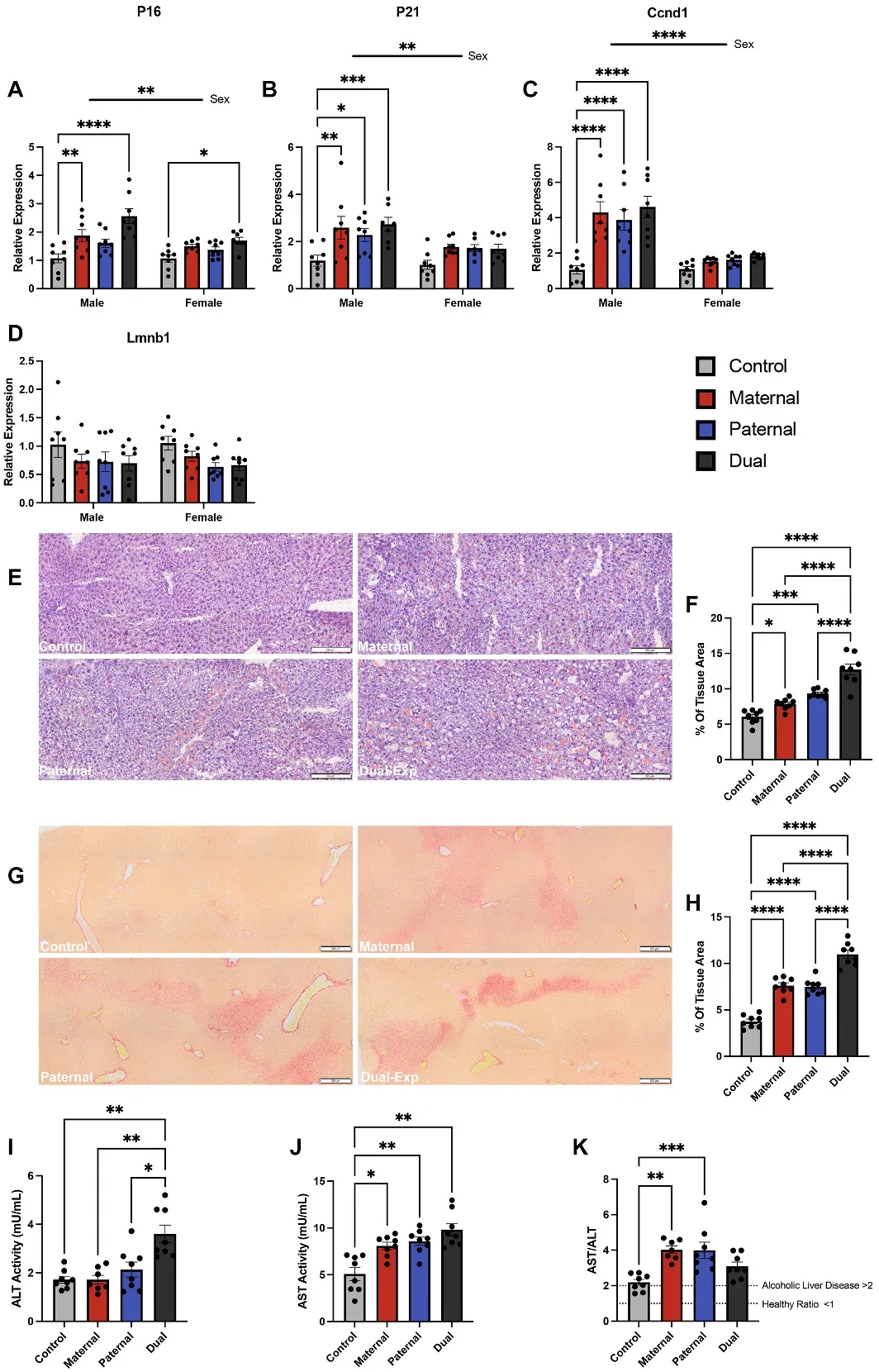
**Maternal, paternal, and dual parental alcohol consumption promote increased hepatic markers of premature cellular senescence and liver disease in male offspring**. RT-qPCR comparison of transcripts encoding (**A**) p16, (**B**) p21^Ink4a^, (**C**) Ccnd1, and (**D**) Lmnb1 between treatments. (**E**) Representative images of male histological sections stained with Oil Red-O. (**F**) Quantification of Oil Red-O staining compared between treatment groups. (**G**) Representative images of male histological sections stained with Picro Sirius red. (**H**) Quantification of Picro Sirius red staining compared between treatment groups. Comparison of (**I**) alanine transaminase (ALT) and (**J**) aspartate transaminase (AST) between treatments. (**K**) Comparison of AST:ALT ratios between treatment groups. We used a two-way ANOVA followed by Tukey's post hoc test to compare treatment groups. Data represent mean ± SEM, n=8, * P < 0.05, ** P < 0.01, *** P < 0.001, **** P < 0.0001.

The incidence of non-alcoholic fatty liver disease (NAFLD) increases with age and correlates with the accumulation of senescent cells, which drive the acquisition of a pro-inflammatory state [61]. This pro-inflammatory state accelerates the onset of aging-related liver disease, including non-alcoholic fatty liver disease, cirrhosis, and fibrosis [62-64]. Using RT-qPCR, we identified increases in transcripts encoding the cell cycle genes p21, p16^Ink4a^, and Ccnd1, but not Lmnb1 across treatments, predominantly in male offspring (Fig. 4A-D). We observed similar transcriptional changes across the kidney, which, like the brain, varied by treatment and sex (Supplementary Fig. 3A-D). In humans, the progression of age-related hepatic dysfunction and disease is significantly influenced by sex, with chronic liver disease more common in men than women [65]. As male offspring exhibited consistent increases in the transcription of senescence-associated markers, we focused on these samples for our histological analysis. In male offspring, we identified significant increases in histological indicators of liver steatosis (Fig. 4E-F) and hepatic fibrosis (Fig. 4G-H). Notably, the histological markers of liver disease are significantly worse in the DualExp group compared to the MatExp and PatExp treatments, suggesting a potential additive effect (Fig. 4F, 4H).

When we assayed clinical markers of liver damage in the male offspring of alcohol-exposed parents, we identified increased alanine transaminase (ALT) levels in DualExp offspring and increased aspartate transaminase (AST) across all three treatments (Fig. 4I-J). In humans, an AST:ALT ratio greater than 2 is strongly suggestive of alcoholic liver disease [66]. We identified a significant increase in the AST:ALT ratio in the MatExp and PatExp treatment groups and, to a lesser extent, in DualExp offspring (Fig. 4K), indicating the aged male offspring of alcohol-exposed parents exhibited clinical signs of alcoholic liver disease, despite never having consumed alcohol themselves. In contrast, we did not observe any differences in ALT, AST, or the AST:ALT ratio in female offspring (Supplementary Fig. 4A-C).

**Figure 5.**
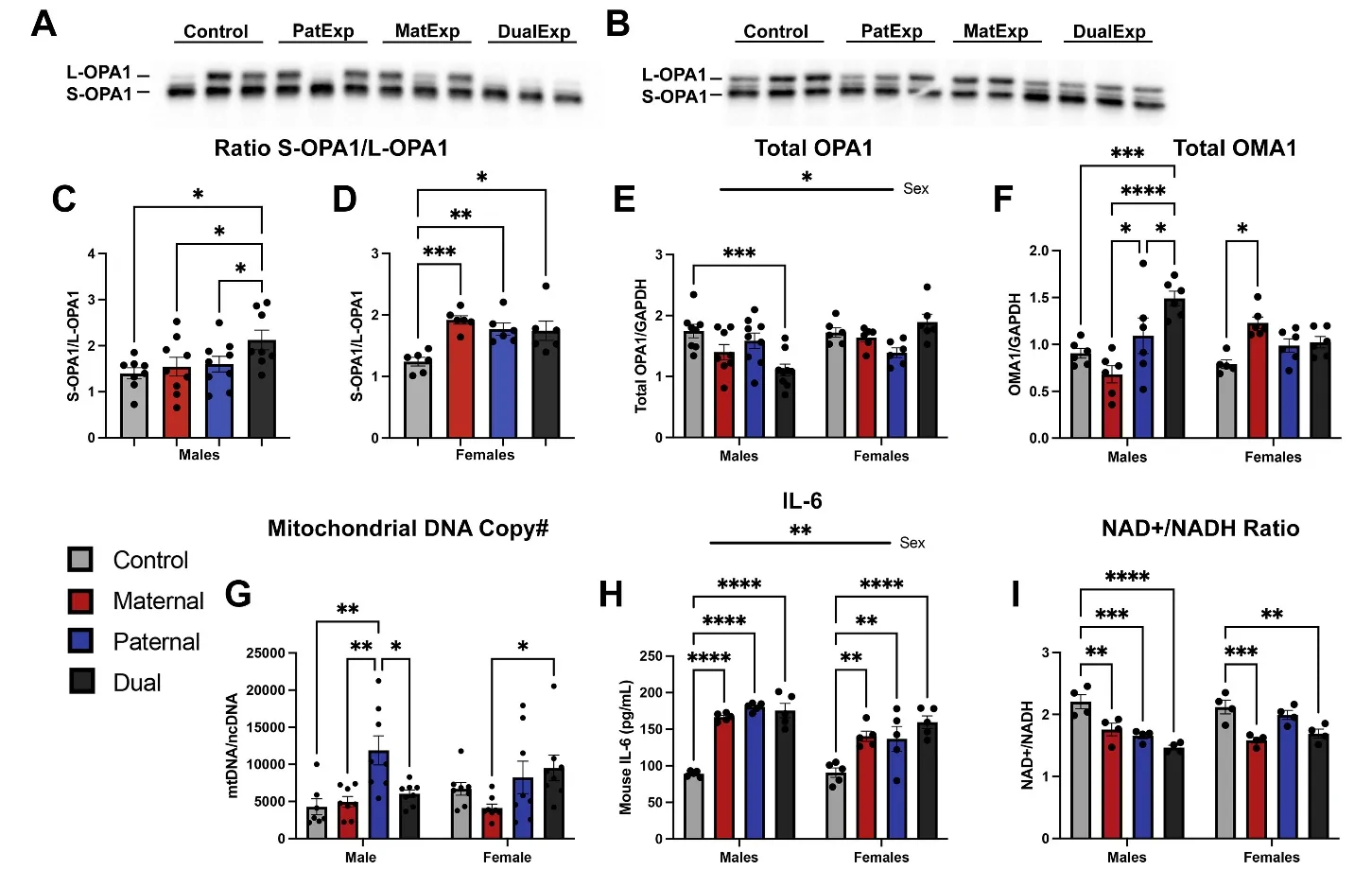
**Maternal, paternal, and dual parental alcohol consumption is associated with lasting adverse effects on offspring mitochondrial function**. Representative Western blot analysis comparing the impacts of parental alcohol exposures on the abundance of the long and short isoforms of OPA1 between liver extracts isolated from (**A**) male and (**B**) female postnatal day 300 offspring. Comparison of the S-OPA1:L-OPA1 ratios between (**C**) male and (**D**) female samples across treatment groups. Comparison of total (**E**) OPA1 and (**F**) OMA1 protein expression between treatments (n=6-9). (**G**) qPCR analysis of mitochondrial DNA copy number (n=8) and (**H**) ELISA quantification of total IL-6 protein (n=5) between treatments. (**I**) Comparison of the NAD+/NADH ratio between treatments (n=4). We used a two-way ANOVA followed by Tukey's post hoc test to compare treatments. Data represent mean ± SEM, * P < 0.05, ** P < 0.01, *** P < 0.001, **** P < 0.0001.

### Stress-induced senescence induced by chronic parental alcohol use correlates with evidence of hepatic mitochondrial dysfunction.

In our previous studies, we have identified transcriptional evidence of mitochondrial dysfunction and an altered mitochondrial stress response in the fetal offspring of alcohol-exposed males [38-40]. During mitochondrial stress, the OMA1 metalloprotease cleaves the long isoform of OPA1 to produce the short S-OPA1 isoform, which promotes increased mitochondrial fission and the release of mitochondrial DNA into the cytoplasm [67]. Likewise, both the proportional abundance of L-OPA1 and total OPA1 expression decline with age [68].

To determine if parental alcohol exposure has a lasting influence on mitochondrial health, we used western blotting to compare the ratio of the long and short isoforms of OPA1 in the offspring's liver. First, we observed higher L-OPA1 variability in male compared to female samples, with some male offspring displaying no detectable L-OPA1 (Fig. 5A-B). When comparing isoform abundance, we identified an increase in the cleaved short form of OPA1 in DualExp male offspring, while notably, females displayed an increased abundance of the short isoform across each of the MatExp, PatExp, and DualExp treatment groups (Fig. 5C-D). In contrast, we only identified a significant decrease in total OPA1 levels in DualExp male offspring (Fig. 5E). In DualExp male offspring, the identified shift in S-OPA1 isoform abundance correlated with increased expression of the OMA1 metalloprotease, while we only identified increased OMA1 in MatExp female offspring (Fig. 5F).

During mitochondrial stress, the release of mitochondrial DNA activates the secretion of inflammatory cytokines, including IL-6, *via* the cGAS-STING pathway [69]. OPA1 loss-of-function also drives the release of inflammatory cytokines and the onset of liver steatosis [68]. Using qPCR, we examined mitochondrial DNA copy number and identified increased abundance only in the PatExp male offspring (Fig. 5G). We also identified increased levels of mitochondrial DNA in PatExp male and female brains but not in the kidney (Supplementary Fig. 5A-B). Strikingly, we observed a significant increase in IL-6 abundance in both male and female offspring across all treatment groups (Fig. 5H). Compromised mitochondrial activity decreases the conversion of cellular NADH to NAD+, reducing the NAD+/NADH ratio [70]. Consistent with our overarching hypothesis of lasting mitochondrial dysfunction, we identified a decreased NAD+/NADH ratio across all treatments in the male and the MatExp and DualExp female liver (Fig. 5I).

**Figure 6.**
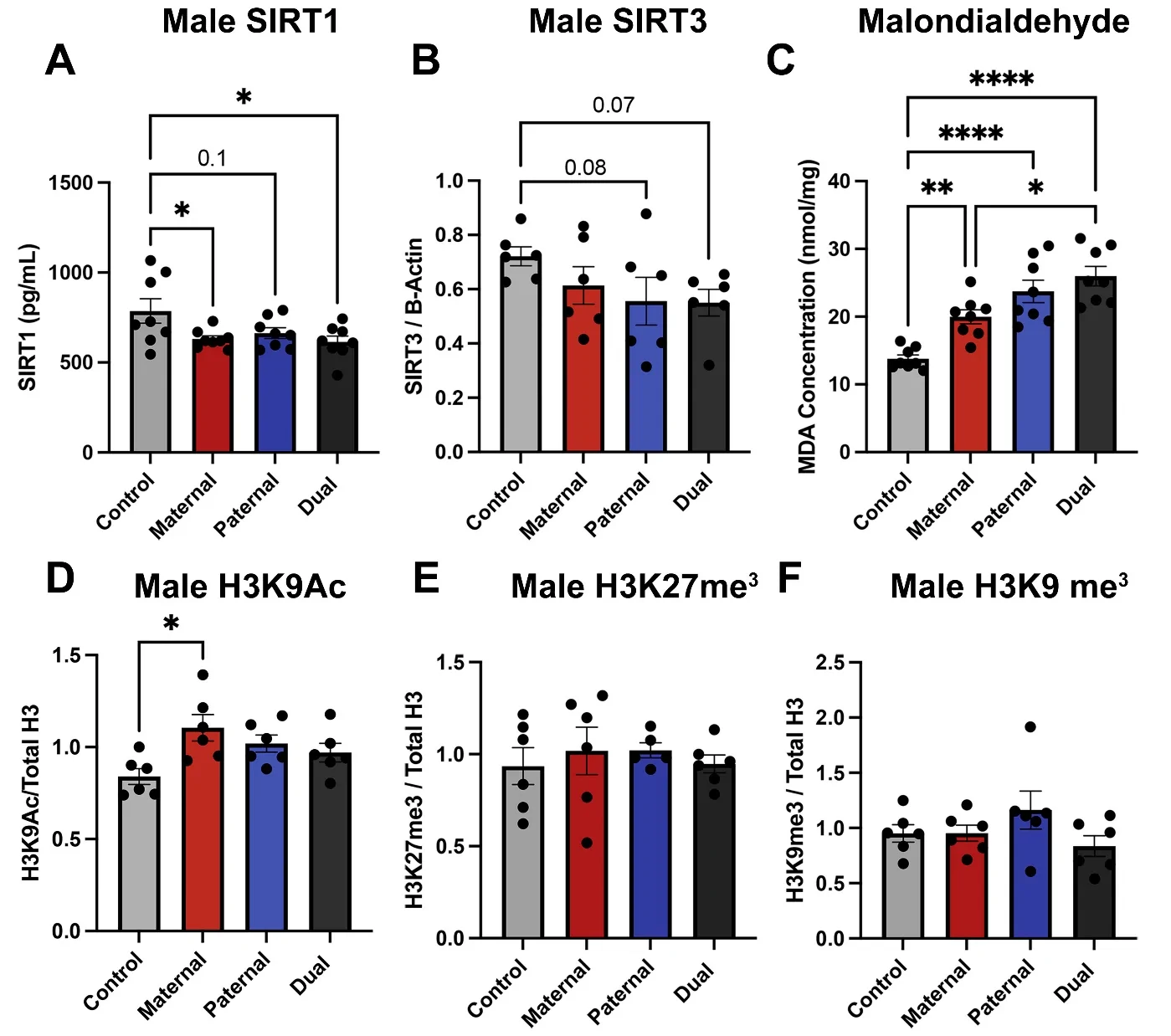
**Offspring of alcohol-exposed parents exhibit decreased Sirtuin protein abundance and increased measures of oxidative damage**. (**A**) ELISA quantification of male SIRT1 protein levels between treatment groups. (**B**) Western blot quantification of SIRT3 protein levels between treatment groups. (**C**) Quantification of cellular malondialdehyde (MDA) levels between treatments using a colorimetric assay. We used Western blotting to compare total (**D**) H3K9Ac, (**E**) H3K27me3, and (**F**) H3K9me3 between treatments. We used a one-way ANOVA followed by Tukey's post hoc test to compare treatments. Data represent mean ± SEM, n=6-8 * P < 0.05, ** P < 0.01, **** P < 0.0001.

Alterations in the cellular NAD+/NADH ratio influence the activity of Sirtuin proteins, including SIRT1 and SIRT3, NAD+-dependent deacetylase involved in the regulation of cellular senescence and aging [71, 72]. Focusing on the male liver, we used an ELISA to quantify SIRT1 abundance. This assay identified decreased SIRT1 abundance in MatExp and DualExp male offspring, while PatExp samples exhibited a more modest decrease (p=0.1) (Fig. 6A). Similarly, we observed decreasing levels of SIRT3 in liver extracts derived from PatExp and DualExp male offspring (p=0.07 and p=0.08) (Fig. 6B). Reduced Sirtuin activity increases cellular vulnerability to oxidative stress, leading to the accumulation of oxidized macromolecules, including DNA, RNA, proteins, and lipids. Consistent with the observed decreases in Sirtuin activity, we detected increased cellular levels of malondialdehyde (MDA) (Fig. 6C), a marker of lipid peroxidation and aging [73]. Decreased SIRT1 activity during aging correlates with increases in histone acetylation, along with changes in H3K27me3 and H3K9me3 [5, 74, 75]. Using western blotting, we observed a significant increase in total H3K9Ac in MatExp male offspring but did not identify any differences in total H3K27me3 or H3K9me3 between treatments (Fig. 6D-F).

## DISCUSSION

Preconception parental and gestational exposures are an emerging area of interest in our efforts to understand the developmental origins of disease and cognitive dysfunction [76, 77]. For example, in humans, periconceptional maternal obesity is associated with an increased risk of age-related diseases, including diabetes, metabolic dysfunction, cardiovascular disease, and cancer [78]. However, studies examining the influences of preconception or early life exposures on adult health almost exclusively emphasize maternal factors, excluding all other considerations, including paternal epigenetic effects [79].

Although very few studies have examined health outcomes in FASD adults, the limited epidemiological studies available demonstrate that the life expectancy of individuals diagnosed with FASDs is approximately 42% shorter than the general population and that they exhibit an increased risk of hospitalizations [20, 80]. Notably, these studies also reveal that individuals with FASDs display an increased incidence of age-related diseases, including cardiovascular disease, metabolic abnormalities (type 2 diabetes, low HDL, and high triglycerides), and liver dysfunction [21-27]. Although preclinical work using mouse models suggests that gestational alcohol exposures increase age-related adiposity and metabolic measures [81], no studies have linked these phenotypes to increased cellular senescence or determined if FASD individuals experience atypical aging, similar to Downs Syndrome [82].

Our previous studies examining fetal offspring derived from alcohol-exposed males or gestational models of alcohol exposure have consistently identified transcriptional alterations in multiple nuclear genes encoding mitochondrial subunits (*Atp5e*, *Atp5l*, and *Ndufa7*), components of the cellular antioxidant response, and transcripts originating from the mitochondrial genome (*mt-Cytb* and *mt-Nd5*) [38-40, 42]. We hypothesized that lasting epigenetic alterations in the transcriptional control of these genes induced by parental drinking would compromise long-term mitochondrial function, resulting in increased markers of premature cellular senescence and an accelerated aging phenotype.

Here, we demonstrate that at midlife (postnatal day 300), MatExp, PatExp, and DualExp adult offspring display increases in established markers of cellular senescence in the brain and liver. In the male offspring, we found that increases in cellular senescence correlated with markers of a pro-inflammatory state and histological evidence of age-associated hepatic fibrosis and liver steatosis. Consistent with our transcriptional analysis of fetal offspring [38-40], we find that aged adult offspring also display evidence of mitochondrial dysfunction, particularly in the liver, where we identified a reduced NAD+/NADH ratio. Based on these observations, we posit that chronic parental alcohol use programs lasting alterations in baseline mitochondrial function. This mitochondrial dysfunction predisposes offspring to accelerated mitochondrial aging and increased stress-induced premature senescence, which is associated with an acceleration of biological aging, particularly age-associated liver disease.

Interestingly, in our analysis of age-related hepatic fibrosis and steatosis in the male offspring of alcohol-exposed parents, we observed additive effects, with measures in DualExp offspring exceeding those caused by either maternal or paternal alcohol use alone. Although these additive effects are not consistent across all the examined markers, our results suggest that, for some alcohol-related phenotypes, maternal and paternal exposures may interact, potentially exacerbating specific outcomes. Our previous studies examining fetal growth at gestational day 16.5 revealed a significant increase in fetal growth restriction in DualExp male offspring but not in the MatExp or PatExp treatments [44]. This early life growth restriction may affect the age-related liver phenotypes we observed here.

While we have never observed additive effects in female offspring per se, several of the alcohol-induced craniofacial abnormalities we identified in our analysis of gestational day 16.5 fetal offspring only appeared in DualExp females, including reductions in the size and spacing of the eyes (biparietal distance) [44]. Therefore, our data reinforce the assertion that paternal drinking may modify alcohol-related phenotypes induced by maternal exposures and emphasize the importance of expanding biomedical studies to examine the potential contributions of both parents. Nonetheless, we still do not understand why we consistently observe sex-specific differences in fetal and adult phenotypes. However, recent clinical studies suggest that sex differences in mitochondrial abundance may sensitize or insulate males and females to certain diseases [83]. This is a current area of focus in our laboratory.

In the mid-2000s, Doug Wallace proposed a bioenergetic perspective could unify many separate complex pathophysiological conditions, including neuropsychiatric diseases such as Alzheimer's and Parkinson's Disease, metabolic diseases such as diabetes and obesity, and age-related diseases, including cognitive decline and cancer [84]. Although predominantly described as a contrast between Mendelian and mitochondrial modes of disease inheritance, Wallace also highlighted that the building blocks of the epigenome (acetylation and methylation, for example) are linked with mitochondrial energy flux [84]. For example, two byproducts of mitochondrial respiration, acetyl-CoA and α-ketoglutarate, are critical substrates in organizing histone structure and nuclear DNA methylation patterns [85]. Theoretically, environmentally-induced alterations in mitochondrial function could disrupt aspects of epigenetic programming, particularly during critical windows of development, leading to heritable (lasting) changes in the nuclear control of mitochondrial gene expression and long-term changes in cellular bioenergetics. Based on our observations, we propose that a bioenergetic perspective may also unify many alcohol-related cognitive, growth, patterning, and disease phenotypes, which may be linked to lasting epigenetic changes in the control of mitochondrial function.

Alcohol exposure potently modifies many aspects of mitochondrial function, resulting in the alteration of the epigenome [86]. These deficits in mitochondrial function appear after both acute and chronic exposures and correlate with alterations in mRNA transcripts encoding mitochondrial genes, altered mitochondrial morphology, and compromised measures of mitochondrial function in the brain, heart, and liver [87]. Here, we demonstrate that periconceptional parental alcohol exposures correlate with lasting alterations in the NAD+/NADH ratio and the emergence of stress-induced premature cellular senescence. Previous studies reveal two modes by which mitochondrial dysfunction influences cellular senescence: one induced by primary mitochondrial dysfunction that relies on AMPK-mediated p53 activation but that is independent of IL-1-activation (MiDAS), and a second involving mitochondria-to-nucleus retrograde signaling that ultimately activates the cytosolic DNA sensing cGAS-STING pathway, stimulating the innate immune system [88, 89]. The previously observed down-regulation of nuclear- and mitochondrial-encoded genes regulating oxidative phosphorylation [38-40] suggests parental alcohol exposures may induce a senescence phenotype aligning with the latter model.

Although the data we present is compelling, there are some limitations to this work. First, we are unable to determine if the changes in mitochondrial function we observed in aged offspring represent a lasting memory of parental alcohol exposure or are another symptom of accelerated aging. For example, although cleavage of OPA1 into the short isoform is an established marker of mitochondrial stress, this ratio also increases with age [67, 68]. Likewise, the NAD+/NADH ratio and SIRT1 levels decline with age. Therefore, we cannot definitively identify the driver of these phenotypes or highlight prospective therapeutic targets.

Second, previous studies demonstrate that paternal experiences can alter maternal-paternal interactions at mating, impacting offspring behavior and growth [90]. As we have yet to measure maternal behaviors, we do not know if male drinking affects maternal nurturing behaviors. Third, we have yet to conclusively identify the epigenetic mechanisms by which the paternal memory of alcohol exposures transmits to offspring or how this change persists across the life course. Our previous studies reveal that alcohol-induced changes in chromatin structure persist after the exposure window, showing that the epigenome carries a memory of this exposure forward in developmental time [91]. However, we do not observe any differences in sperm DNA methylation, and none of the observed changes in sperm histone post-translational modifications mapped to genes regulating bioenergetics or mitochondrial function [36, 92]. Therefore, we do not see evidence to support the direct epigenetic inheritance of mitochondrial bioenergetics from sperm to offspring.

Previous work in worms demonstrates that parental mitochondrial stress exerts dose-dependent effects on offspring phenotypes, which transmit to the next generation via germline noncoding RNAs [93-95]. Similarly, work using mouse models reveals that a range of paternal stressors also alters sperm small noncoding RNAs, which correlate with changes in offspring behavior, growth, and metabolism [96-101]. Notably, emerging studies demonstrate that some sperm noncoding RNAs derive from the mitochondrial genome and that alterations in these epigenetic signals correlate with premature activation of embryonic oxidative metabolism, with long-term impacts on metabolic outcomes [102]. We and others also observe alcohol-induced enrichment of sperm small noncoding RNAs interfacing with antioxidant pathways [35, 37, 103]. These observations suggest alcohol-induced paternal mitochondrial stress drives changes in sperm-inherited noncoding RNAs, which induce lasting deficits in offspring mitochondrial health. This topic is an area of active research in our lab.

Alcohol-induced alterations in mitochondrial morphology and function within fetal tissues are a widely described symptom in FASD research [87]. Notably, our studies demonstrate the emergence of similar phenotypes in the offspring of alcohol-exposed males, demonstrating that these phenotypes are programmed outcomes and not the consequence of direct alcohol exposure. If our preclinical studies translate to humans, FASD individuals may inherit a heightened risk for programmed mitochondrial dysfunction and an early onset of age-related diseases. Further, this population may also benefit from therapeutic interventions supporting NAD+ metabolism [70]. Finally, these data reinforce the suggestion that we must expand public health messaging surrounding alcohol use and FASDs to include men and caution couples on the potential cumulative dangers of parental alcohol use.

## Supplementary material

The Supplementary data can be found online at: www.aginganddisease.org/EN/10.14336/AD.2024.0722.

## Data Availability

Data sharing is not applicable to this article as no new data were created or analyzed in this study.
