# Supplementary material for: Parental Alcohol Exposures Associate with Lasting Mitochondrial Dysfunction and Accelerated Aging in a Mouse Model
Source: Aging Dis. 2024 Jul 20;16(4):2408–25. doi: 10.14336/AD.2024.0722 (PMC12221405; doi:10.14336/AD.2024.0722)
Supplement: Supplementary file 1 — The Supplementary data can be found online at: www.aginganddisease.org/EN/10.14336/AD.2024.0722. [file AD-16-4-2408-s.pdf]

SUPPLEMENTARY DATA

Parental Alcohol Exposures Associate with Lasting Mitochondrial Dysfunction and Accelerated Aging in a Mouse Model

Alison Basel, Sanat S. Bhadsavle, Katherine Z. Scaturro, Grace K. Parkey, Matthew N. Gaytan, Jai J. Patel, Kara N. Thomas, Michael C. Golding

© 2024. Basel A et al. Published online at http://www.aginganddisease.org/EN/10.14336/AD.2024.0722

SUPPLEMENTARY DATA

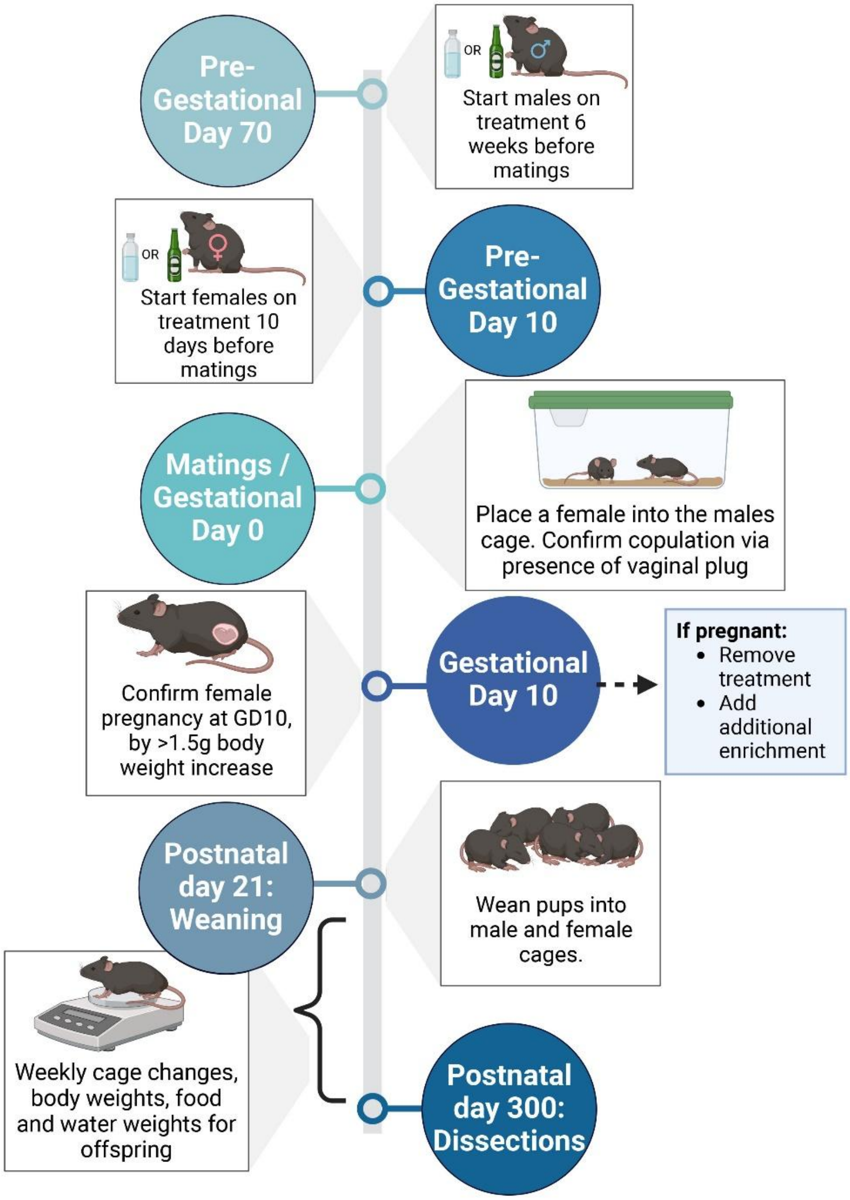

Supplementary Figure 1. Experimental flowchart depicting the timeline of parental alcohol exposures, mouse breeding, gestation, and offspring assessments.

© 2024. Basel A et al. Published online at http://www.aginganddisease.org/EN/10.14336/AD.2024.0722

SUPPLEMENTARY DATA

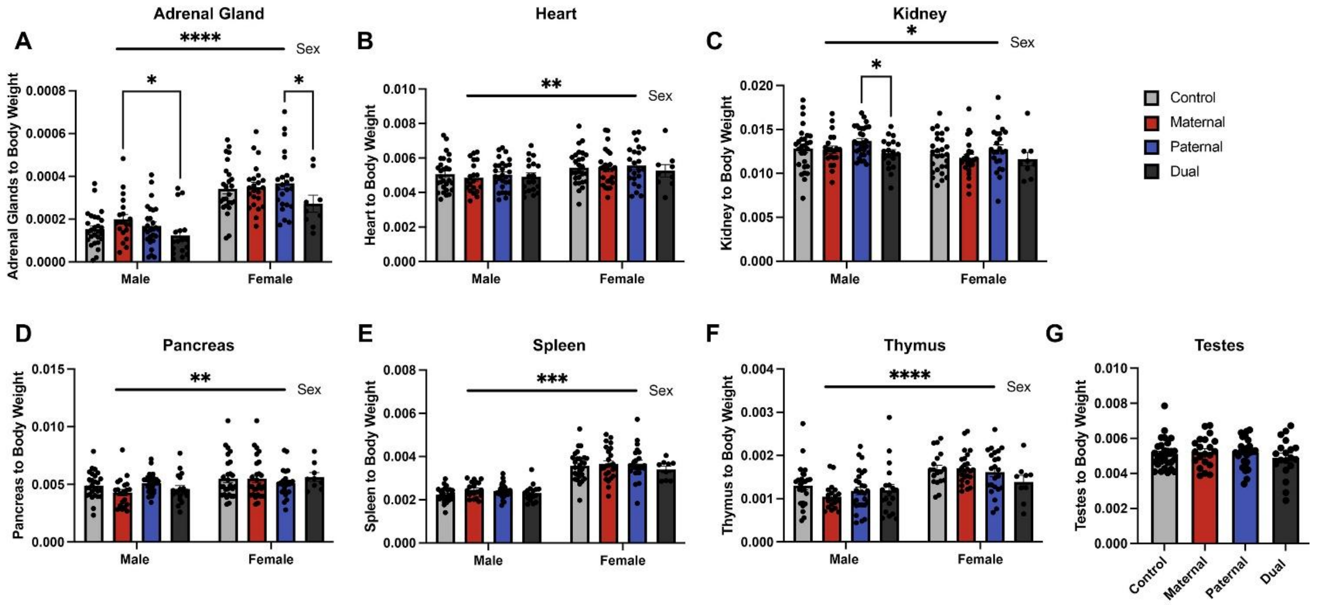

Supplementary Figure 2. Maternal, paternal, and dual parental alcohol consumption exert sex- and treatment-specific effects on offspring normalized organ weights. We compared bodyweight-normalized **(A)** adrenal, **(B)** heart, **(C)** kidney, **(D)** pancreas, **(E)** spleen, **(F)** thymus, and **(G)** testis weights between the treatment groups. We analyzed datasets using a two-way ANOVA followed by Tukey's post hoc test. Data represent mean ± SEM, (n=9-29) * $P < 0.05$, ** $P < 0.01$, *** $P < 0.001$, **** $P < 0.0001$.

© 2024. Basel A et al. Published online at http://www.aginganddisease.org/EN/10.14336/AD.2024.0722

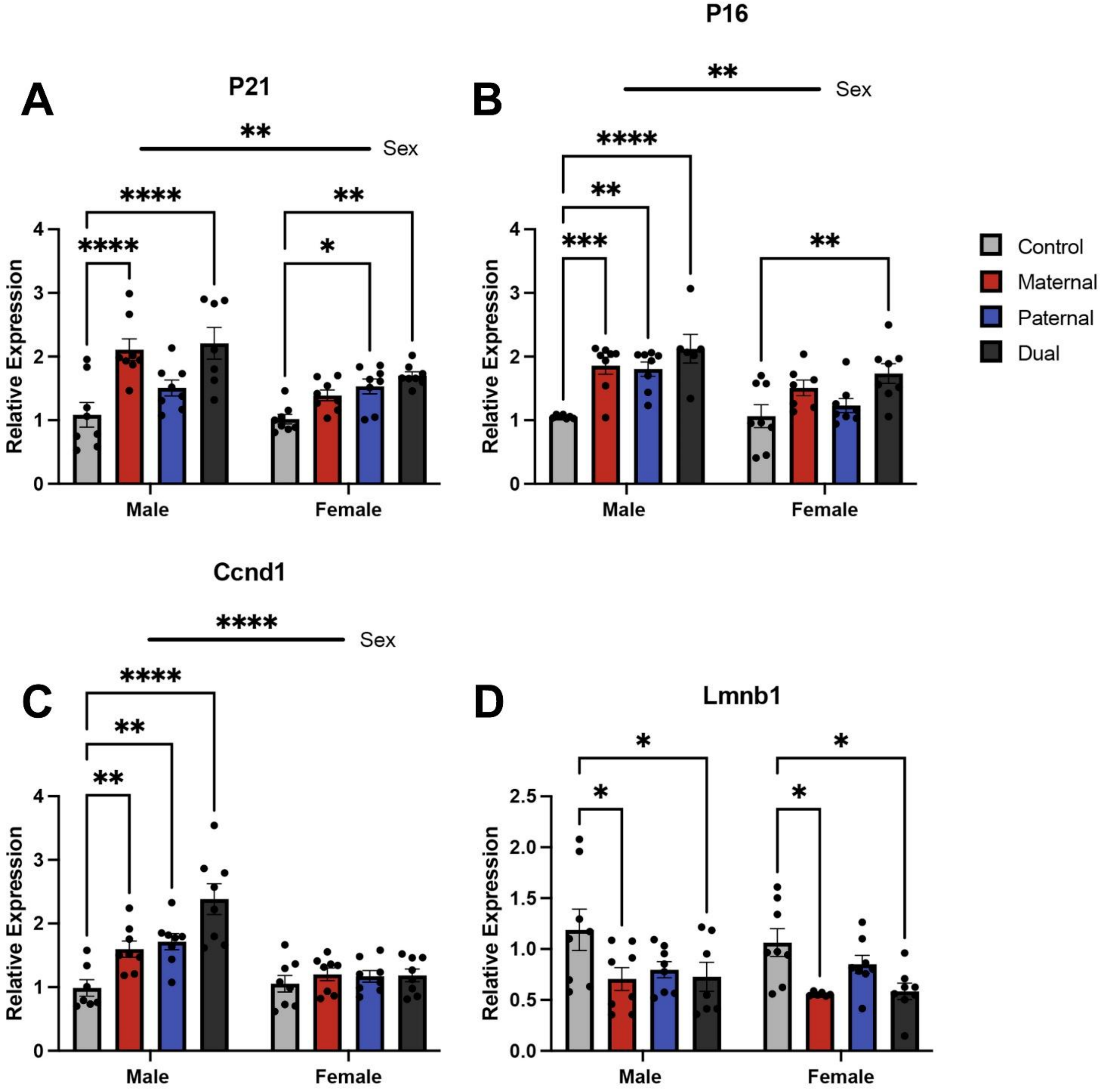

Supplementary Figure 3. Maternal, paternal, and dual parental alcohol consumption induce markers of premature cellular senescence in the postnatal day 300 offspring kidney. We used reverse transcriptase quantitative polymerase chain reaction (RT-qPCR) analysis to compare transcripts encoding **(A)** p16, **(B)** p21Ink4a, **(C)** Cyclin D1 (Ccnd1), and **(D)** *Lamin-B1* (Lmnb1) between treatments. We used a two-way ANOVA followed by Tukey's post hoc test to compare treatment groups. Data represent mean ± SEM, (n=8) * $P < 0.05$, ** $P < 0.01$, *** $P < 0.001$, **** $P < 0.0001$.

© 2024. Basel A et al. Published online at http://www.aginganddisease.org/EN/10.14336/AD.2024.0722

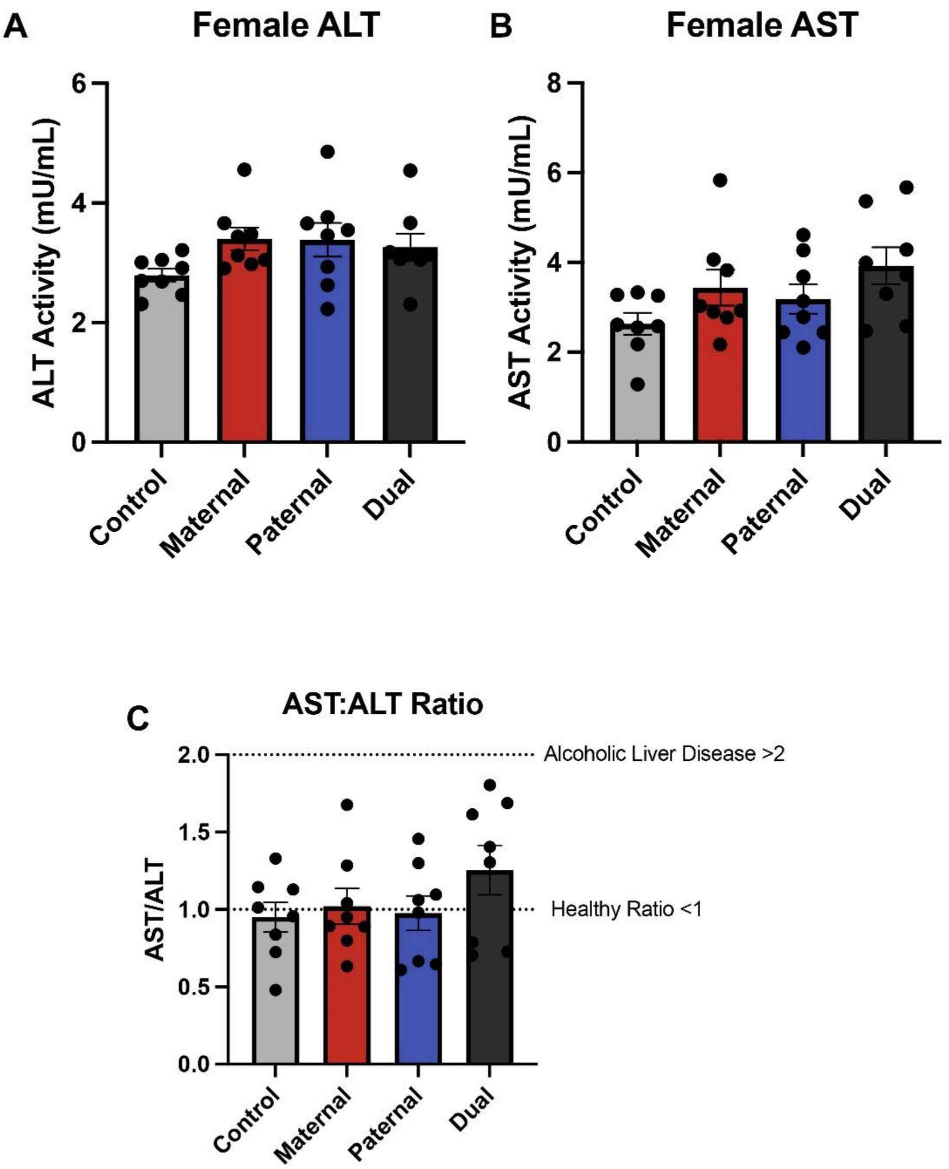

Supplementary Figure 4. Analysis of clinical markers of liver damage in the female offspring of alcohol-exposed parents. Comparison of **(A)** alanine transaminase (ALT) and **(B)** aspartate transaminase (AST) between treatments. **(C)** Comparison of AST:ALT ratios between treatment groups. We used a two-way ANOVA to compare treatment groups. Data represent mean ± SEM, (n=8).

© 2024. Basel A et al. Published online at http://www.aginganddisease.org/EN/10.14336/AD.2024.0722

SUPPLEMENTARY DATA

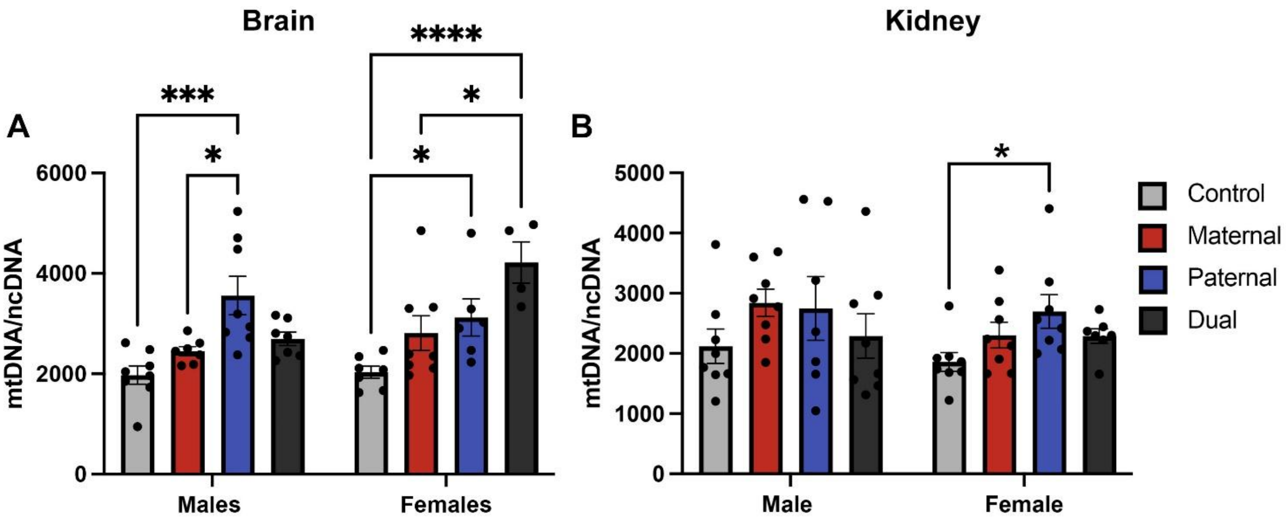

Supplementary Figure 5. Maternal, paternal, and dual parental alcohol consumption induce treatment-specific changes in mitochondrial DNA copy number within the postnatal brain and kidney. We used quantitative polymerase chain reaction (qPCR) to measure mitochondrial DNA copy number between the postnatal day 300 **(A)** brain and **(B)** kidney between treatment groups and analyzed the data using a two-way ANOVA followed by Tukey's post hoc test to compare treatment groups. Data represent mean ± SEM, (n=8) * P < 0.05, *** P < 0.001, **** P < 0.0001.

Supplementary Table 1. Descriptions of the sample sizes and statistical tests for each figure.

**Litter and sex information per treatment group**					
**Treatment**		**Number of Litters**	**Number of Males**		**Number of Females**
**Control**		11	28		27
**Maternal**		10	29		22
**Paternal**		12	21		25
**Dual**		9	17		10
**Graph**		**Statistical Test**	**Sample Size**		**Outliers**
Figure 1: A multiplex mouse model to study the impacts of parental drinking on offspring senescence and age-related phenotypes					
**B:**	Sire body weight	Two-way ANOVA, multiple comparisons using Sidak.	n =	16 control<br>15 ethanol	0
**C-D:**	Average daily dose of EtOH	One-way ANOVA, multiple comparisons using Tukeys, or Unpaired t test	C: n =<br><br>D: n =	19 paternal<br>17 maternal preconception<br>22 maternal gestation<br><br>11 paternal<br>8 dual	0
**E-F:**	Maternal daily dose and food intake	Two-way ANOVA, multiple comparisons using Sidak.	n =	13 preconception control<br>17 preconception ethanol<br>20 gestation control<br>20 gestation ethanol	2 preconception control<br>1 gestation control
**G-H:**	Daily calories and weight gain	Unpaired t test.	G: n =	22 control<br>20 ethanol	0
			H: n =	22 control<br>20 ethanol	

© 2024. Basel A et al. Published online at http://www.aginganddisease.org/EN/10.14336/AD.2024.0722

SUPPLEMENTARY DATA

**I-J:**	Gestation length and litter size	Kruskal-Wallis, multiple comparisons using Dunn's.	I: n =	11 control<br>12 maternal<br>11 paternal<br>8 dual	I: 1 control	
			J: n =	10 control<br>12 maternal<br>11 paternal<br>8 dual		
**K:**	Sex ratio	Chi-Square analysis followed by Fisher's Exact test for individual comparisons.	n =	55 control<br>51 maternal<br>46 paternal<br>27 dual	0	
Figure 2: Parental alcohol exposures induce sex- and treatment-specific effects on offspring lean weight and normalized organ weights						
**A-B:**	Body weight analysis	Two-way ANOVA, multiple comparisons using Uncorrected Fisher's LSD.	a: n =	28 control<br>21 maternal<br>29 paternal<br>19 dual	0	
			b: n =	27 control<br>25 maternal<br>23 paternal<br>9 dual		
**C-G:**	DEXA scan analysis	Two-way ANOVA, multiple comparisons using Sidak.	Males: n =	10 control<br>6 maternal<br>11 paternal<br>11 dual	D:	1 paternal female
			Females: n =	9 control<br>8 maternal<br>8 paternal<br>9 dual	F:	1 paternal male<br>1 maternal female<br>1 dual female
**H-I:**	Organ to body weight	We inserted organ weights into Excel, then divided by total body weight.<br>Two-way ANOVA, multiple comparisons using Tukey.	Males: n =	28 control<br>21 maternal<br>29 paternal<br>19 dual	I:	1 maternal male<br>1 control female
			Females: n =	27 control<br>25 maternal<br>23 paternal<br>9 dual		
Figure 3: Increased markers of cellular senescence in the brains of offspring derived from alcohol-exposed parents						
**B:**	B-gal quantification	Two-way ANOVA, multiple comparisons using Tukey.	Males: n =	6 control<br>6 maternal<br>6 paternal<br>6 dual	0	
			Females: n =	6 control<br>6 maternal<br>6 paternal<br>5 dual		
**C-F:**	Senescent genes qPCR	Two-way ANOVA, multiple comparisons were done to the control group using Dunnett.	Males: n =	8 control<br>8 maternal<br>8 paternal<br>7 dual	C:	1 maternal male<br>1 paternal male<br>1 control female<br>1 maternal female

© 2024. Basel A et al. Published online at http://www.aginganddisease.org/EN/10.14336/AD.2024.0722

SUPPLEMENTARY DATA

			Females: n =	8 control<br>8 maternal<br>8 paternal<br>8 dual	E-F:	1 control female
Figure 4: parental alcohol exposures program cumulative effects on the male offspring's predisposition to develop senescence-associated liver disease						
**A-D:**	Senescent genes qPCR	Two-way ANOVA, multiple comparisons were done to the control group using Dunnett.	Males: n =	8 control<br>8 maternal<br>8 paternal<br>8 dual	B:	1 dual male<br>1 paternal female
			Females: n =	8 control<br>8 maternal<br>8 paternal<br>8 dual		
**F-H:**	Histology quantification	Ordinary One-way ANOVA, multiple comparisons using Tukeys.	n =	8 control<br>8 maternal<br>8 paternal<br>8 dual	0	
**I-K:**	Liver function tests	I&K: Ordinary One-way ANOVA, multiple comparisons using Tukeys.<br>J: Kruskal-Wallis, multiple comparisons using Dunn's.	n =	8 control<br>8 maternal<br>8 paternal<br>8 dual	I:<br>K:	1 maternal<br>1 maternal
Figure 5: Stress-induced senescence induced by chronic parental alcohol use correlates with evidence of hepatic mitochondrial dysfunction						
**C-D:**	S/OPA1-L/OPA1	Ordinary One-Way ANOVA, multiple comparisons using Fisher's LSD.	Males: n =	9 control<br>9 maternal<br>9 paternal<br>9 dual	C:	1 control<br>1 dual
			Females: n =	6 control<br>6 maternal<br>6 paternal<br>6 dual		
**E:**	Total OPA1	Two-way ANOVA, multiple comparisons were done to the control group using Dunnett.	Males: n =	9 control<br>9 maternal<br>9 paternal<br>9 dual	1 control male<br>1 maternal male	
			Females: n =	6 control<br>6 maternal<br>6 paternal<br>6 dual		
**F:**	Total OMA1	Two-way ANOVA, multiple comparisons using Tukey.	Males: n =	6 control<br>6 maternal<br>6 paternal<br>6 dual	0	
			Females: n =	5 control<br>6 maternal<br>6 paternal<br>6 dual		
**G:**	Mt copy number	Two-way ANOVA, multiple comparisons using Tukey.	Males: n =	8 control<br>8 maternal<br>8 paternal<br>8 dual	1 control female	
			Females: n =	8 control<br>8 maternal<br>8 paternal		

© 2024. Basel A et al. Published online at http://www.aginganddisease.org/EN/10.14336/AD.2024.0722

SUPPLEMENTARY DATA

				8 dual	
**H:**	ELISA IL-6	Two-way ANOVA, multiple comparisons using Tukey.	Males: n =	5 control<br>5 maternal<br>5 paternal<br>5 dual	0
			Females: n =	5 control<br>5 maternal<br>5 paternal<br>5 dual	
**I:**	NAD/NADH ratio	Two-way ANOVA, multiple comparisons were done to the control group using Dunnett.	Males: n =	4 control<br>4 maternal<br>4 paternal<br>4 dual	0
			Females: n =	4 control<br>4 maternal<br>4 paternal<br>4 dual	
Figure 6: Offspring of alcohol-exposed parents exhibit decreased Sirtuin protein abundance and increased measures of oxidative damage					
**A:**	ELISA Sirt1	Ordinary One-way ANOVA, multiple comparisons were done to the control group using Dunnett.	n =	8 control<br>8 maternal<br>8 paternal<br>8 dual	0
**B:**	SIRT3 quantification	Ordinary One-Way ANOVA, multiple comparisons using Fisher's LSD.	n =	6 control<br>6 maternal<br>6 paternal<br>6 dual	0
**C:**	MDA assay	Ordinary One-way ANOVA, multiple comparisons using Tukeys	n =	8 control<br>8 maternal<br>8 paternal<br>8 dual	0
**D:**	H3K9Ac quantification	Ordinary One-way ANOVA, multiple comparisons using Tukeys	n =	6 control<br>6 maternal<br>6 paternal<br>6 dual	0
**M-N:**	H3K27me3 and H3K9me3 quantification	Ordinary One-Way ANOVA, multiple comparisons using Fisher's LSD.	n =	6 control<br>6 maternal<br>6 paternal<br>6 dual	1 paternal
Supplemental Figure 2: Normalized organ weights					
**A:**	Adrenal gland	Two-way ANOVA, multiple comparisons using Uncorrected Fisher's LSD.	Males: n =	28 control<br>21 maternal<br>29 paternal<br>19 dual	Males: 1 paternal, 2 dual<br><br>Females: 1 control, 1 maternal
			Females: n =	27 control<br>25 maternal<br>23 paternal<br>9 dual	
**B:**	Heart	Two-way ANOVA, multiple comparisons using Tukeys.	Males: n =	28 control<br>21 maternal<br>29 paternal<br>19 dual	Males: 1 control, 2 maternal<br><br>Females: 0
			Females: n =	27 control<br>25 maternal<br>23 paternal<br>9 dual	
**C:**	Kidney	Two-way ANOVA, multiple comparisons using Uncorrected Fisher's LSD.	Males: n =	28 control<br>21 maternal<br>29 paternal	Males: 0<br><br>Females: 0

© 2024. Basel A et al. Published online at http://www.aginganddisease.org/EN/10.14336/AD.2024.0722

SUPPLEMENTARY DATA

				19 dual	
			Females: n =	27 control<br>25 maternal<br>23 paternal<br>9 dual	
**D:**	Pancreas	Two-way ANOVA, multiple comparisons using Tukeys	Males: n =	28 control<br>21 maternal<br>29 paternal<br>19 dual	Males: 1 control<br><br>Females: 0
			Females: n =	27 control<br>25 maternal<br>23 paternal<br>9 dual	
**E:**	Spleen	Two-way ANOVA, multiple comparisons using Tukeys.	Males: n =	28 control<br>21 maternal<br>29 paternal<br>19 dual	Males: 1 control, 1 maternal, 1 paternal, 2 dual<br><br>Females: 0
			Females: n =	27 control<br>25 maternal<br>23 paternal<br>9 dual	
**F:**	Thymus	Two-way ANOVA, multiple comparisons using Tukeys.	Males: n =	28 control<br>21 maternal<br>29 paternal<br>19 dual	Males: 2 control, 2 maternal<br><br>Females: 4 control
			Females: n =	27 control<br>25 maternal<br>23 paternal<br>9 dual	
**G:**	Testes	Ordinary One-way ANOVA, multiple comparisons using Tukeys	n =	28 control<br>21 maternal<br>29 paternal<br>19 dual	2 paternal
Supplemental Figure 3: RT-qPCR to compare senescent transcripts in the kidney					
**A-D:**	Senescent genes qPCR	Two-way ANOVA, multiple comparisons were done to the control group using Dunnett.	Males: n =	8 control<br>8 maternal<br>8 paternal<br>7 dual	B: 1 maternal female<br><br>C: 1 control male<br><br>D: 2 maternal males
			Females: n =	8 control<br>8 maternal<br>8 paternal<br>8 dual	
Supplemental Figure 4: Female ALT and AST liver analysis					
**A-D:**	ALT and AST analysis	Ordinary One-Way ANOVA, multiple comparisons Tukeys	n =	8 control<br>8 maternal<br>8 paternal<br>8 dual	0
Supplemental Figure5: Mitochondrial copy number in the brain and kidney					
**A:**	mtDNA/ ncDNA analysis in brain	Two-way ANOVA, multiple comparisons using Tukeys.	Males: n =	8 control<br>8 maternal<br>8 paternal<br>7 dual	Males: 1 maternal<br><br>Females: 1 control
			Females: n =	8 control<br>8 maternal<br>8 paternal<br>8 dual	
**B:**	mtDNA/ ncDNA analysis in kidney	Two-way ANOVA, multiple comparisons using Tukeys.	Males: n =	8 control<br>8 maternal<br>8 paternal<br>7 dual	Males: 1 paternal<br><br>Females: 1 dual
			Females: n =	8 control	

© 2024. Basel A et al. Published online at http://www.aginganddisease.org/EN/10.14336/AD.2024.0722

				8 maternal<br>8 paternal<br>8 dual	

Supplementary Table 2: Sequence information for the PCR primers.

Gene	Forward	Reverse
β-actin	CCACCATGTACCCAGGCATT	CGGACTCATCGTACTCCTGC
α-tubulin	CTGATGTATGCCAAGCGTGC	TCGCCTTCCACAGAATCCAC
P21(WAF/Cip1)	GTTCCTTGCCACTTCTTACCT	GGTGAGTCCTAACTGCCATCC
P16Ink4a	CGCTGGGTGGTCTTTGTGTA	GCTCTGCTCTTGGGATTGGC
CCND1	TGCGTGCAGAAGGAGATTGT	CTTCTTCAAGGGCTCCAGGG
LMNB1	ATCAACCAATGGTGGTCTT	TCCTCGGGTATGGTGGTCTT
D-Loop3	TCCTCCGTGAAACCAACAA	AGCGAGAAGAGGGGCATT
Tert	CTAGCTCATGTGTCAAGACCCTCTT	GCCAGCACGTTTCTCTCGTT

© 2024. Basel A et al. Published online at http://www.aginganddisease.org/EN/10.14336/AD.2024.0722
